# Intestinal biopsy is not always required to diagnose celiac disease: a retrospective analysis of combined antibody tests

**DOI:** 10.1186/1471-230X-13-19

**Published:** 2013-01-23

**Authors:** Annemarie Bürgin-Wolff, Buser Mauro, Hadziselimovic Faruk

**Affiliations:** 1 Institute for Celiac disease, Bahnhofplatz 11, 4410, Liestal, Switzerland; 2 Statistik Dr. M. Buser, Riehen, Switzerland

## Abstract

**Background:**

The objective of this study was to compare celiac disease (CD)– specific antibody tests to determine if they could replace jejunal biopsy in patients with a high pretest probability of CD.

**Methods:**

This retrospective study included sera from 149 CD patients and 119 controls, all with intestinal biopsy. All samples were analyzed for IgA and IgG antibodies against native gliadin (ngli) and deamidated gliadin peptides (dpgli), as well as for IgA antibodies against tissue transglutaminase and endomysium.

**Results:**

Tests for dpgli were superior to ngli for IgG antibody determination: 68% vs. 92% specificity and 79% vs. 85% sensitivity for ngli and dpgli, respectively. Positive (76% vs. 93%) and negative (72% vs. 83%) predictive values were also higher for dpgli than for ngli. Regarding IgA gliadin antibody determination, sensitivity improved from 61% to 78% with dpgli, while specificity and positive predictive value remained at 97% (*P* < 0.00001). A combination of four tests (IgA anti-dpgli, IgG anti-dpgli, IgA anti- tissue transglutaminase, and IgA anti-endomysium) yielded positive and negative predictive values of 99% and 100%, respectively and a likelihood ratio positive of 86 with a likelihood ratio negative of 0.00. Omitting the endomysium antibody determination still yielded positive and negative predictive values of 99% and 98%, respectively and a likelihood ratio positive of 87 with a likelihood ratio negative of 0.01.

**Conclusion:**

Antibody tests for dpgli yielded superior results compared with ngli. A combination of three or four antibody tests including IgA anti-tissue transglutaminase and/or IgA anti- endomysium permitted diagnosis or exclusion of CD without intestinal biopsy in a high proportion of patients (78%). Jejunal biopsy would be necessary in patients with discordant antibody results (22%). With this two-step procedure, only patients with no CD-specific antibodies would be missed.

## Background

Celiac disease (CD) is an immune-mediated enteropathy that is caused by intolerance to gluten in genetically susceptible individuals. Its prevalence among the European population is approximately 1% [1,2], and is even higher among the elderly [3]. Thus, CD is one of the most frequently occurring lifelong diseases. Serological tests to diagnose CD have improved substantially in the last 20 years. In 1998 [4], we proposed a low-risk and cost-effective algorithm to diagnose various forms of gluten-sensitive enteropathy that achieved a positive predictive value (ppv) of 99%, using a combination of different antibody determinations: anti-endomysium (EMA), IgA anti-tissue transglutaminase (IgA anti-tTG), and IgA and IgG anti-native gliadin (IgA and IgG anti-ngli). In a population with a high pretest probability of disease, synchronous determination of three or four CD-specific antibodies has a very high ppv and negative predictive value (npv), and may eliminate the necessity of small-bowel biopsy in many patients suspected of having CD [4].

In recent years, the use of ngli as an antigen in antibody-detection tests has been replaced with deamidated gliadin peptides (dpgli), which perform better diagnostically than ngli [5-12]. Our goal in this study was to investigate whether using dpgli instead of ngli, alone or in combination with other tests (EMA and IgA anti-tTG), reduces the number of jejunal biopsies without missing CD patients during the diagnostic procedure.

## Methods

### Patients

Included in this retrospective study were serum samples from 268 patients on a gluten-containing diet. The samples were collected in hospitals or medical services throughout Switzerland, Germany and Austria. The sera were then sent to the Institute for Coeliac Disease in Liestal, Switzerland, where the antibody determinations were performed without any knowledge of each patient’s clinical condition. All patients from whom we received a jejunal histology report and clinical data were included in the study. At the beginning of our studies, all patients with symptoms suggestive of CD underwent small intestinal biopsy; antibody determinations were performed at the same time. This diagnostic procedure gradually changed with time as serological tests gained increasing importance in diagnosis, and for an undefined period patients were sometimes selected for biopsy when IgA anti tTG or EMA were positive. The patients suffered from gastrointestinal symptoms such as diarrhea, constipation, poor weight gain, chronic vomiting, abdominal pain, flatulence, and failure to thrive; or disorders such as unexplained weight loss in adults, iron-deficiency anemia, lassitude, psychiatric disorders, short stature, and diabetes type 1. IgA-deficient CD patients were excluded. Serum for antibody determinations was obtained within 2 months before or one month after endoscopic intervention.

### Sample analysis

All samples were analyzed for antibodies against tTG, ngli, and dpgli by fully automated fluoroenzyme immunoassay tests (Elia Celikey IgA, Elia Gliadin IgA, Elia Gliadin IgG, Elia Gliadin DP IgA and Elia Gliadin DP IgG; Phadia [now Thermo Fisher Scientific], Freiburg, Germany) performed on the Phadia 100 instrument in accordance with the manufacturer’s instructions. With the help of ROC curves, we calculated the optimal cut-off values for our sample. For all analyzes except IgA anti-dgpli, we found the best sensitivity and specificity to be consistent with the recommendations of the respective manufacturer. For IgA anti- dpgli. we found that using a cut off value of 7 instead of 10 resulted in somewhat higher sensitivity (78% instead of 71%) while maintaining the same specificity.

For each antigen, we determined the best cut-off value within our sample with respect to the sum of false-positive and false-negative results: IgA anti tTG = 7, IgA anti-ngli= 7, IgG anti-ngli = 7, IgA anti-dpgli = 7, and IgG anti-dpgli = 10. EMA was analyzed by indirect immunofluorescence on monkey esophagus sections: cut-off = serum dilution: 1:5.

### Statistical analysis

In addition to the usual descriptors for diagnostic tests, such as sensitivity, specificity, and positive and negative predictive values, the quantity “efficiency” was used: the efficiency of a diagnostic test is its percentage of correct outcomes. Because predictive values are dependent on the prevalence of the disease in the study population, we also calculated the positive and negative likelihood ratios for CD, which are independent of the pretest probability of disease. The likelihood ratio is the ratio of the probability that a patient with the disease has a particular test result divided by the probability that a patient without the disease has the same test result. Contingency tables were evaluated using “Fisher’s Exact Test”. To test whether the replacement or addition of a diagnostic test improves the outcome to a statistically significant degree, McNemar’s test for significant changes was applied. Both tests were used in their precise form (not only asymptotic) as available in the software package StatXact version 6.3.0. (Cytel Software Corporation Cambridge, MA, USA).

## Results

The histology of 149 consecutive patients (104 females, age range at biopsy 0.9–80 years, median age 29 years; 45 males, age range at biopsy 2–73 years, median age 13 years) revealed subtotal or complete villous atrophy, hyperplasia of the crypts, and an increase in intraepithelial lymphocytes (Marsh classification 3a, b, or c lesions). All of these patients recovered after starting a gliadin-free diet and were regarded as having CD. The biopsies of 119 consecutive patients (66 females, age range at biopsy 1.5–72 years, median age 17 years; 53 males, age range at biopsy 2–66 years, median age 7 years) revealed a normal mucosa or a mucosa with slight, nonspecific changes; these patients were considered free of CD and served as controls (The Control group was younger than the group with CD, P=0.0074).

### Deamidated gliadin peptides compared with native gliadins as antigens

Sera from 149 patients with CD and 119 control patients were tested for IgG and IgA antibodies against dpgli and ngli proteins. IgG antibody determination for dpgli was superior to that for ngli. Specificity was 68% vs. 92% and sensitivity was 79% vs. 85% for ngli and dpgli, respectively; ppv was 76% vs. 93% and npv was 72% vs. 83% for ngli and dpgli, respectively. For IgA antibody determination, sensitivity was 61% vs. 78% for ngli and dpgli, respectively, while the specificity and ppv remained at a high level of 97% (McNemar’s test for significant changes *P* < 0.00001, Table 1). Because dpgli antigens were clearly superior to ngli, we used only dpgli for further CD-specific antibody determinations.

**Table 1:** Antibody tests against deamidated gliadin (dpgli) vs. native gliadin (ngli) in 149 CD patients and 119 controls

	**IgA anti-ngli**	**IgA anti-dpgli**	**IgG anti-ngli**	**IgG anti-dpgli**
Sensitivity	61%	78%	79%	85%
Specificity	97%	97%	68%	92%
Positive predictive value	97%	97%	76%	93%
Negative predictive value	67%	78%	72%	83%

McNemar’s test for significant changes *p* < 0.00001.

61 correct changes; 154 remain correct; 7 wrong changes; 46 remain wrong.

### Antibody profile in CD and control patients

We also determined the levels of IgA anti-tTG and EMA in sera from the 149 CD patients and 119 controls (Table 2). Because the IgA anti-tTG and EMA results were comparable, we have omitted the EMA results; instead, we have shown the IgA anti- tTG, and IgA anti-dpgli, and IgG anti-dpgli antibody levels of each individual and compared them with the histological result. We used a multiple test consisting of three individual tests, which produce a total of eight possible results. We defined the outcome of the multiple tests as positive only when all three individual tests were above the cut-off, and as negative only when all three individual tests were below the cut-off. The majority of the patients (208/268) had either positive (110) or negative (98) results in all three tests. Nearly all patients (109/110) who tested positive for antibodies in all three tests had CD according to histological findings. The ppv was 99% in our population, with a CD frequency of 59% (Table 3). Patients who did not test positive for CD-specific antibodies in any of the three tests were almost all free of CD according to the results of jejunal biopsy (96/98 patients); the npv was 98%. Patients with discordant antibody results (60/268 patients, 22%) could not be defined as positive or negative for CD with the multiple tests and remained unclassified. The likelihood positive ratio (lr+) was 87 and the likelihood ratio negative (lr-) was 0.01 (Table 3). These findings indicate that a biopsy is avoidable if all antibody values are either above or below the cut-off. In patients with discordant antibody results, an intestinal biopsy is necessary to diagnose or exclude CD.

**Table 2:** Antibody profile in each of 149 CD patients and 119 controls

**IgA anti- tTG**	**IgA anti- dpgli**	**IgG anti- dpgli**	**CD n = 149**	**Controls n = 119**	**Total n = 268**	**Classification**
**+**	**+**	**+**	**109**	**1**	**110**	**110 positives**
						
+	+	-	7	1	8	
+	-	+	15	4	19	
+	-	-	13	10	23	60 not
-	+	+	0	0	0	classified
-	+	-	0	2	2	
-	-	+	3	5	8	
						
**-**	**-**	**-**	**2**	**96**	**98**	**98 negatives**

+ antibody present; - antibody absent.

**Table 3:** Performance of single antibody tests and selected combinations n = 268, 149 CD patients and 119 controls

	**fn**	**fp**	**nc**	**sens**	**spec**	**ppv**	**npv**	**effic**	**lr+**	**lr-**
**Single tests**				**%**	**%**	**%**	**%**	**%**		
IgA anti-dpgli	33	4	0	78	97	97	78	86	23	0.23
Iga anti-dpgli	22	10	0	85	92	93	83	88	10	0.16
IgA anti-tTG	5	16	0	97	87	90	95	92	7	0.04
EMA	3	18	0	98	85	89	97	98	6	0.02
**Combinations of 2 tests** IgG anti-dpgli +										
IgA anti-tTG	2	5	39	83	82	96	98	83	20	0.04
IgG anti-dpgli + EMA	1	6	39	84	82	95	99	83	17	0.01
IgA anti-dpgli + IgG anti-dpgli	15	1	37	73	89	99	88	80	87	0.11
**Combinations of 3 tests** IgA anti-dpgli +										
IgG anti-dpgli + EMA	1	1	62	72	81	99	99	76	86	0.01
IgA anti-dpgli + IgG anti-dpgli + IgA anti-tTg*	*2*	*1*	*60*	*73*	*81*	*99*	*98*	*76*	*87*	*0.01*
IgG anti-dpgli + EMA + IgA anti-tTg	0	5	45	83	80	96	100	81	20	0.00
**Combination of 4 tests** IgG anti-dpgli +										
IgA anti-dpgli + EMA + IgA anti-tTG	0	1	65	72	79	99	100	75	86	0.00

### Performance of single antibody tests and selected test combinations

We compared the performance of IgA anti-dpgli, IgG anti-dpgli, IgA anti-tTG, and EMA tests, and calculated the sensitivity, specificity, ppv, npv, lr+, lr-, and efficiency of each test and some of the possible test combinations (Table 3). We also indicated the absolute number of patients whose antibody test results were falsely positive or falsely negative for CD, as well as those who could not be classified based on antibody tests. Most of the following diagnostic tests are multiple tests (compare with Table 2). We defined the outcome of a multiple test as positive only when all individual tests were above the cut-off, and as negative only when all individual tests were below the cut-off. Test combinations containing only IgA antibodies were not considered; they are unsuitable for diagnostic purposes, because of the possibility that some patients may be deficient in IgA.

Currently, biopsies are often performed when a patient’s IgA anti-tTG or EMA test is positive. Negative serological results are usually not followed by a jejunal intervention, unless there is a very strong clinical suspicion of CD. However, the data in Table 3 clearly show that single tests are neither specific nor sensitive enough to reduce the number of biopsies in patients with symptoms of CD, although the number of nonclassified patients was zero. Single tests such as the widely used IgA anti-tTG test can give rise to many falsely classified patients.

A combination of two tests also yielded many incorrectly classified patients and is therefore unsuitable for reducing the number of biopsies. The two-test combinations yielded either too many false-positive diagnoses (IgG anti-dpgli + IgA anti-tTG or IgG anti-dpgli + EMA) or too many false-negative diagnoses (IgA anti-dpgli + IgG anti- dpgli), although the number of nonclassified patients was smaller than in combinations with more than two tests. The combination of four tests was optimal: only one patient was falsely positive, no patients were falsely negative, the ppv was 99%, the npv was 100%, the lr+ was 86, and the lr- was 0.00 For practical reasons, a combination of three tests using IgA anti-tTG instead of EMA in combination with IgA anti-dpgli and IgG anti-dpgli (Table 2) may be sufficient to set a standard (ppv 99%, one false-positive result; npv 98%, two false-negative results; and lr+ = 87, lr- = 0.01). A biopsy was avoidable in 208/268 patients (78%), while 60/268 patients (22%) could not be diagnosed with the combination of serological tests because their results were in disagreement (only one or two results above the cut-off, with the remaining result(s) below the cut-off; Table 2).

## Discussion

The diagnosis of CD has traditionally depended upon intestinal biopsies and has been extended to include an array of serological markers. The guidelines of the European and North American societies for gastroenterology require a biopsy for diagnosis [13,14]. Recently, the European Society for Pediatric Gastroenterology, Hepatology, and Nutrition published guidelines allowing the diagnosis of CD without a biopsy in some situations [15]. CD is usually diagnosed when the duodenal and jejunal mucosa display villous atrophy, crypt hyperplasia, and an increase in intraepithelial lymphocytes [16-19]. However, different diseases not related to gluten- sensitive enteropathy can induce a flat mucosa, thus mimicking CD. Moreover, patients with gluten-sensitive enteropathy and normal small bowel mucosal architecture have also been described [20-24]. Most likely because of a lack of technical proficiency with grasping biopsy forceps or endoscopic procedure, biopsy specimens have been shown to be sufficient for diagnosis of CD in only 90% of cases [25]. Furthermore, CD may be missed during histological examinations owing to variations in different pathologists’ assessments [26]. Because of this, and because of the inconvenience and high cost associated with jejunal biopsy and the high prevalence of CD in the general population, less-invasive tests are required. In the last 20 years, serological tests for the diagnosis of CD have improved substantially [27-33]. For practical and ethical reasons, patients with negative serology sometimes did not undergo a biopsy unless clinical indications of CD were evident. This procedure causes a verification bias because the gold standard (histology of the mucosa) is not always available for negative tests [29]. On the other hand, a positive test result demanded a biopsy even when there was only a slight clinical suspicion of CD. Today, it is nearly impossible to overcome this bias for ethical reasons; therefore, the bias may be present in many studies.

The data contained in Table 3, however, indicate that the criteria for choosing the best tests must be defined. For clinicians who want to reduce the number of jejunal interventions in a population with a high frequency of CD, the best test is the one with the lowest sum of false-positive and false-negative diagnoses: the test with the highest ppv, the highest npv, a high likelihood ratio positive, and a low likelihood ratio negative. In our study, a combination of four antibody tests yielded a ppv of 99%, an npv of 100%, an lr+ of 86, and an lr- of 0.00. For practical reasons, we may omit EMA from our combination of antibody tests, and instead chose the test combination of IgG anti-dpgli + IgA anti-dpgli + IgA anti-tTG (Tables 2 and 3), with a ppv of 99%, an npv of 98%, an lr+ of 87, and an lr- of 0.01, as the first step in our diagnostic procedure. Out of 268 patients, 208 (78%) were correctly classified with these serological tests: they had either three tests above or three tests below the cut- off. Intestinal biopsy was necessary as a second diagnostic step in the remaining 60 patients (22%), who had discordant antibody results. This two-step diagnostic procedure reduces the number of intestinal biopsies and increases the sensitivity of the entire diagnostic procedure; only CD patients without any CD-specific antibodies would be missed.

In 1998 [4], we suggested the above combination of serological tests as a low-risk and cost-effective algorithm for diagnosing various forms of CD. This combination — still using anti ngli — was confirmed in a total of 1,873 patients with jejunal biopsy [30-32]. Because of patient preselection according to their symptoms, the prevalence of CD was 59%. The ppv of three tests with congruent positive antibody results was >99%. The npv of all three antibody tests was 98%. However, 37% (599/1,873) of the patients with discordant antibody results could not be classified by antibody tests alone. In the present study, the number of nonclassified patients was reduced from 37% to 22% (*P* < 0.001) because anti-dpgli performed better than anti-ngli. Thus, in a population with a high pretest probability of CD, using a combination of three or four antibody tests should obviate the need for as many as 78% of jejunal biopsies.

Several recent studies have questioned the necessity of performing a jejunal biopsy on all individuals with suspected CD [34-39]. One approach defined five criteria, including clinical signs, four of which had to be fulfilled for a diagnosis of CD [38]. Other studies describe the association of very high IgA anti-tTG antibody titers with Marsh 3 histopathology [35-37]. Therefore, recent guidelines, released by the European Society for Pediatric Gastroenterology, Hepathology, and Nutrition stated that intestinal biopsy is redundant in patients with high anti-tTG antibody titers (>10 times the upper limit of normal) [15]. These proposals attain a high specificity and will result in few patients receiving a false-positive diagnosis. However, many CD patients do not have such high anti-tTG titer, and will therefore require intestinal biopsy. Sugai et al. [39] investigated single antibody tests and various combinations of two-antibody tests in populations with different pretest probabilities for CD. As in our study, sensitivity was lower in combined tests than in single tests; however, ppv increased significantly not only in the population with high pretest probability for CD, but also in the group with low pretest probability for CD. They concluded that: “Appropriate use of CD serology might accurately identify the vast majority of CD patients in populations with different pretest probabilities”.

Recently, Vermeersch et al. illustrated the utility of likelihood ratios for the interpretation of CD serology. [40] The likelihood ratio for CD was much higher for double positive test than for single positive test results. [40] Our results showed comparable test results for single and double positive analyses. (Table 3) Similarly, triple positive tests had a high likelihood ratio. However, the best test for CD exclusion was the triple negative test which had a significantly lower likelihood ratio than the double negative test reults reported by Vermeersch et al. (p=0.000037).

Therefore, we speculate that the combined tests with the very high likelihood ratio positive and the very low likelihood ratio negative achieved in the present study group will also identify patients in populations with a low CD frequency.

## Conclusion

There is no single test — not even jejunal biopsy — that can conclusively diagnose or exclude CD in every individual. Therefore, we propose the following two-step diagnostic procedure: The first step is the combined, simultaneous determination of IgA anti-dpgli and IgG anti-dpgli + IgA anti-tTG and/or EMA. The vast majority of patients will have either three positive or three negative results, obviating the need for a biopsy. The second step, jejunal biopsy, should be performed only in patients with discordant antibody results (i.e., in patients whose CD status cannot be classified by antibody tests alone). In any case, effects of a gluten-free diet must be controlled.

## Competing interest

The authors declare that they have no competing interests.

## Authors’ contributions

Study design: ABW and FH; Patient selection: ABW and FH; Sample analysis and interpretation: ABW and FH; Statistics: MB; Manuscript writing: ABW, MB, and FH. All authors have read and approved the final manuscript.

## Pre-publication history

The pre-publication history for this paper can be accessed here:

http://www.biomedcentral.com/1471-230X/13/19/prepub
